# Impacts of tirzepatide on older patients with diminished β‐cell function and dementia

**DOI:** 10.1111/ggi.70018

**Published:** 2025-02-25

**Authors:** Toshitaka Sawamura, Shigehiro Karashima, Ai Ohmori, Mitsuhiro Kometani, Takashi Yoneda

**Affiliations:** ^1^ Department of Internal Medicine Asanogawa General Hospital Kanazawa Japan; ^2^ Department of Health Promotion and Medicine of the Future Kanazawa University Kanazawa Japan

## Abstract

Tirzepatide could control postprandial hyperglycemia even in cases with diminished β‐cell function. This finding gives new insight into the treatment of older patients with diabetes. However, tilzepatide does not replace basal insulin in cases with diminished β‐cell function and requires careful observation for sarcopenia.

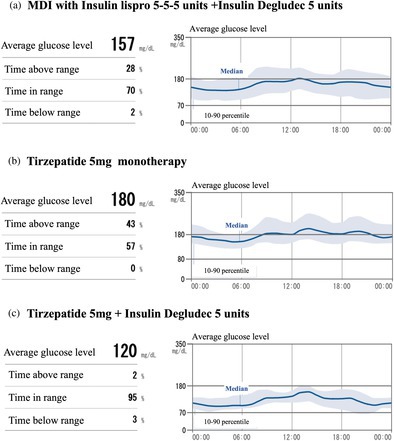

Dear Editor,

Recently, we encountered a case of an 81‐year‐old male with type 2 diabetes with diminished β‐cell function and dementia. His glycemic control significantly improved following the administration of tirzepatide by nurses during daytime care services.

The patient was diagnosed with type 2 diabetes at the age of 62. He commenced a regimen of multiple daily insulin injections (MDI) utilizing insulin glargine (Gla) and insulin lispro (Lis), owing to β‐cell dysfunction. Initially, his Hemoglobin A1c (HbA1c) levels were maintained between 6.5% and 7.5% with MDI. However, over the past 3 years, as his dementia progressed, his HbA1c levels rose to approximately 10%. Following this deterioration, his treatment regimen was modified from MDI to the treatment of dulaglutide combined with Gla; however, satisfactory glycemic control was not achieved. Consequently, the regimen was adjusted to twice‐daily insulin degludec (Deg)/aspart (Asp) with metformin and vildagliptide at the dose of 22 units, 1500 mg, and 100 mg per day, respectively. Nevertheless, self‐insulin injection became difficult owing to the progression of dementia, exacerbated by inadequate cooperation from his wife. Therefore, the patient was referred to our hospital and subsequently admitted for comprehensive diabetes management.

Upon admission, his body mass index was 22.3 kg/m^2^, and his HbA1c level was 9.3%. MDI with Lis and Deg was introduced, and his blood glucose levels as evaluated with intermittently scanned continuous glucose monitoring (isCGM) improved (Fig. 1a). After the stabilization of his blood glucose level, β‐cell function was evaluated by glucagon loading test and urine test. C‐peptide immunoreactivities measured before and after glucagon stimulation were 0.17 and 0.48 ng/mL, respectively. Additionally, urine C‐peptide excretion was 15 μg/day, indicating impairment in insulin secretion. However, self‐insulin injection at home was deemed impractical, necessitating his transfer to a facility providing continuous nursing support for the ongoing management of MDI, despite his strong desire to return home. Consequently, MDI was tried to switch to tirzepatide monotherapy, which could be administrated by a nurse during daytime care services. Tirzepatide was initially administered at a dosage of 2.5 mg and subsequently escalated to 5 mg over a 4‐week use of 2.5 mg. While tirzepatide 5 mg per week effectively suppressed postprandial hyperglycemia, it failed to control fasting hyperglycemia (Fig. 1b). Therefore, the treatment of tirzepatide combined with Deg was implemented, and the patient was subsequently discharged to home. Post‐discharge, Deg and tirzepatide were administered by a nurse during daytime services 5 days a week, and by his wife on the remaining 2 days. Oral antidiabetic agents were not prescribed owing to the patient's inability to manage medications independently. This treatment successfully controlled fasting and postprandial hyperglycemia, achieving a time in range (TIR) of 95% (Fig. 1c). No adverse effects, regarding gastrointestinal symptoms and severe hypoglycemia, were observed. This patient was classified category II, with a target HbA1c range set between 7.0 and 7.9%, according to the guideline.^1^ His outpatient HbA1c levels ranged from 6.8 to 7.1%. Although this HbA1c range was slightly below the recommended target range, the TIR remained well controlled at 95%, significantly exceeding the target range of 50% in older patients.^2^ The dosage of Deg was reduced after this isCGM evaluation owing to a time below range of 3%, slightly above the goal of 1% in older patients.^2^ The isCGM was subsequently discontinued, and no further data were available after the insulin dosage adjustment. Rehabilitation was provided during daytime care services to maintain muscle strength, and no excessive weight loss was observed. Six months after initiating tirzepatide, the patient experienced a modest weight reduction of 0.6 kg, a decrease of 2 kg in right‐hand grip strength, and a 3 kg increase in left‐hand grip strength. While these changes were minor, they were deemed clinically insignificant, particularly within the context of routine clinical practice, and it was concluded that tirzepatide administration did not induce sarcopenia.

**Figure 1:**
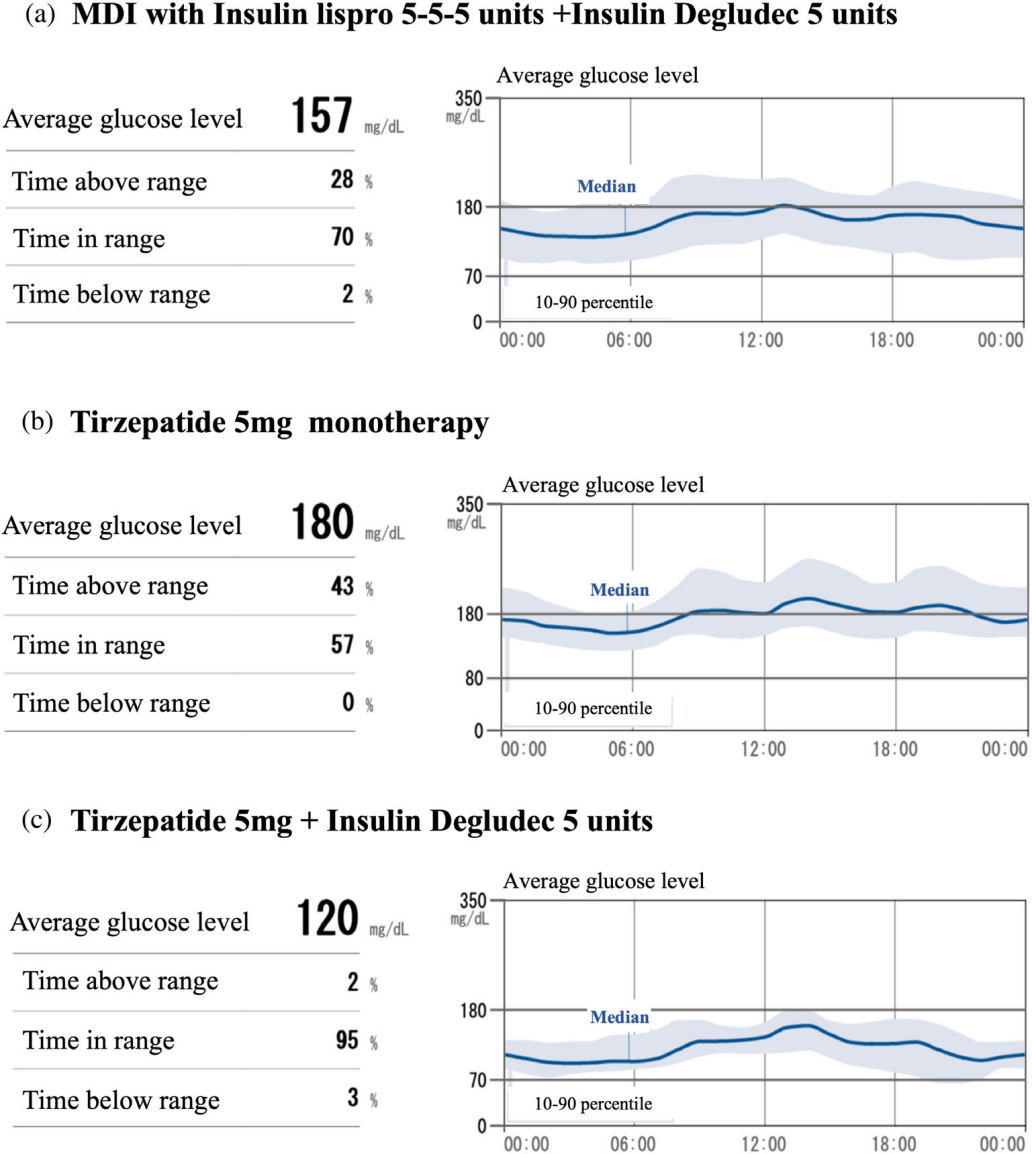
Findings of intermittently scanned continuous glucose monitoring. (a) The data with insulin lispro 15 units and degludec 5 units. (b) The data with tirzepatide 5 mg monotherapy. (c) The data with tirzepatide 5 mg and insulin degludec 5 units.

Tirzepatide is a novel once‐weekly subcutaneous antidiabetic agent that functions as a dual agonist for glucose‐dependent insulinotropic polypeptide (GIP) and glucagon‐like peptide‐1 (GLP‐1) receptors,^3^ demonstrating superior blood glucose‐lowering effects compared with dulaglutide.^4^ Given that the efficacy of GLP‐1 analogs is contingent upon the preservation of β‐cell function, their effectiveness is anticipated to be limited in scenarios characterized by significantly impaired β‐cell function. The C‐peptide index (CPI) serves as a crucial predictor for the successful transition from MDI to basal‐supported prandial therapy (BPT) with liraglutide, and a CPI greater than 1.103 ng/dL is requisite for achieving HbA1c levels below 7.0%.^5^ However, GIP can enhance postprandial insulin secretion twofold compared with GLP‐1.^6^ Furthermore, tirzepatide significantly suppresses postprandial glucagon secretion in comparison to dulaglutide.^7^ These foundational data affirm that the efficacy of tirzepatide may effectively control postprandial hyperglycemia in cases exhibiting diminished β‐cell function. Omura *et al*. reported on four cases of older patients treated with tirzepatide that resulted in adequate blood glucose control.^8^ In one of these cases, a patient who had been treated with Asp three times daily was able to discontinue Asp following the initiation of tirzepatide. This case had several similarities to the present case. However, the report did not provide detailed information regarding β‐cell function. In the current case, Lis could be discontinued with the administration of tilzepatide, despite substantial laboratory evidence of impaired β‐cell function. This finding offers novel insight into the efficacy of tirzepatide in patients with compromised β‐cell function.

Dementia and polypharmacy are characteristics of older patients with diabetes.^9^ However, treatment adherence declines as dementia progresses. Thus, simplification of treatment is desirable. Compared with MDI, a combination of tirzepatide and basal insulin is simple and could improve treatment adherence and blood glucose control in older patients. Furthermore, the burden on caregivers is also a major problem in the treatment of diabetes in older patients. The decrease in the number of insulin injections performed by the patient's wife was considered useful in the current case, as this reduced the burden on her.

Thirzepatide has the potential to facilitate body weight reduction in addition to lowering blood glucose levels.^4^ Weight reduction is also observed upon transitioning from GLP‐1 analogs and is not influenced by baseline characteristics.^10^ In young obese individuals with diabetes, weight reduction could contribute to glycemic control through the amelioration of fatty liver.^11^ However, sarcopenia is associated with the incidence of all‐cause mortality in elderly patients with diabetes.^12^ Consequently, meticulous monitoring of body weight and grip strength is imperative and maintenance of muscle strength through rehabilitation is essential.

In conclusion, tirzepatide may effectively mitigate postprandial glycemic elevations even in patients with compromised β‐cell function and dementia. Further evidence is anticipated to be accumulated on the administration of tilzepatide to older patients.

## Disclosure statement

All authors declare that they have no conflict of interest.

## Author contributions

TS and AO are the attending physicians of this patient. TS wrote the manuscript. AO provided the figure legend. SK, MK, and TY supervised manuscript writing.

## Funding

This work did not receive any specific grant.

## Patient consent statement

Written informed consent was obtained from the patient and his wife.

## Data Availability

Data sharing not applicable no new data generated.
